# Atypical group 1 neuraminidase pH1N1-N1 bound to a group 1 inhibitor

**DOI:** 10.1007/s13238-015-0197-6

**Published:** 2015-09-03

**Authors:** Ying Wu, Christopher J. Vavricka, Yan Wu, Qing Li, Santosh Rudrawar, Robin J. Thomson, Mark von Itzstein, George F. Gao, Jianxun Qi

**Affiliations:** CAS Key Laboratory of Pathogenic Microbiology and Immunology, Institute of Microbiology, Chinese Academy of Sciences, Beijing, 100101 China; Institute for Glycomics, Griffith University, Gold Coast Campus, Nathan, QLD 4222 Australia

**Dear Editor,**

Influenza virus is a member of the *Orthomyxoviridae* family and is the causative agent of seasonal and pandemic flu. There are three types of influenza virus: A, B and C. Influenza A viruses possess the greatest host-range and reassortment ability and all known influenza pandemics have been caused by type A. Influenza A viruses contain 8 negative-strand RNA segments, encoding 14 proteins, including 3 major surface proteins, hemagglutinin (HA), neuraminidase (NA) and the M2 proton channel. Due to their accessibility on the surface of the virus, HA, NA and M2 have been the primary targets for the design of anti-influenza therapeutics (Du et al., 2012).

NA is a retaining sialidase that catalyzes hydrolysis of the ketosidic bond of terminally linked sialic acid residues, thereby functioning as the influenza receptor-destroying element. As a surface enzyme that can be targeted by transition state analogues, NA is currently the most successful anti-influenza drug target with several successful drugs commercially available or under clinical trials (von Itzstein, 2007; Moscona, 2008; Vavricka et al., 2013).

There are currently 9 known subtypes of functional influenza A NA based upon antigenicity. These 9 subtypes can be categorized into two phylogenetic groups with N1, N4, N5 and N8 in group 1, and N2, N3, N6, N7 and N9 in group 2 (Wu et al., 2014). Based on earlier crystal structures of N2 (Colman et al., 1983) and N9 (Webster et al., 1987), structure-based inhibitors, like zanamivirs and oseltamivir, were developed. In 2006, the first group 1 NA crystal structures of N1, N4 and N8 were solved and they all contained an additional cavity adjacent to the active site, the 150-cavity, which is formed by an open conformation of the 150-loop (residues 147–151) (Russell et al., 2006). Since then, novel inhibitors targeting this extra cavity have been pursued, with 3-(*p*-tolyl)allyl-Neu5Ac2en (Rudrawar et al., 2010) as a good example of such an inhibitor. This compound demonstrated specificity for inhibition of group 1 over group 2 NAs (N1 NA: *K*_i_ 2 µmol/L; N2 NA: *K*_i_ 220 µmol/L) and selective inhibition of the growth of N1-containing viruses compared with an N2-containing virus in plaque reduction assays (Rudrawar et al., 2010).

Previously we reported that the 2009 pandemic H1N1 influenza virus (pH1N1) NA (09N1) does not have a 150-cavity and therefore it was unclear whether or not group 1 specific inhibitors which access the 150-cavity could be effective against it (Li et al., 2010). We proposed that the I149V mutant may play a role in the unique 150-loop properties of 09N1. Meanwhile, it has been reported that group 1 specific inhibitor 3-(*p*-tolyl)allyl-Neu5Ac2en does indeed work against 09N1 (Rudrawar et al., 2010). Therefore in this study we prepared 09N1 with and without the I149V substitution and soaked 3-(*p*-tolyl)allyl-Neu5Ac2en into both forms. We found that 3-(*p*-tolyl)allyl-Neu5Ac2en bound both proteins in a similar conformation, but distinct from the canonical group 1 members N8 and N5. In contrast, 3-(*p*-tolyl)allyl-Neu5Ac2en could not be soaked into the group 2 member, N2, but rather the inhibitor was found to bind the second binding site (Kobasa et al., 1997) of group 2 member, N3, further confirming the inability of group 2 NA to bind 3-(*p*-tolyl)allyl-Neu5Ac2en.

NA proteins were produced using a baculovirus expression system as described previously (Li et al., 2010). 09N1, N2, N3 and N5 are from A/California/04/2009 (H1N1), A/RI/5+/1957(H2N2), A/Swine/Missouri/2124514/2006 (H2N3) and A/duck/Alberta/60/1976 (H12N5), respectively. 09N1-I149V mutant was generated by using primer F: 5′-CATTCCAATGGAACCGTTAAAGACAGGAGC-3′ and primer R: 5′-GCTCCTGTCTTTAACGGTTCCATTGGAATG-3′. 3-(p-tolyl)allyl-Neu5Ac2en was produced according to the reported methods.

The protein crystals were grown according to our previous reports (Li et al., 2010). 09N1-I149V crystals were obtained in exactly the same condition as 09N1 (Li et al., 2010). All crystals were first incubated in mother liquor containing 5–20 mmol/L inhibitor for 2–3 h and then flash-cooled at 100 K. Diffraction data were collected at KEK beamline BL1A or SSRF beamline BL17U. The data were processed and scaled by using the HKL-2000 software program. Data collection and processing statistics are summarized in Table S1. All the NA complex structures with 3-(*p*-tolyl)allyl-Neu5Ac2en were solved by molecular replacement using Phaser from the CCP4 program suite.

The structure of 09N1-I149V mutant still displayed a closed 150-loop as did that of 09N1 (Fig. 1A and 1B, respectively). Earlier work showed that a 09N1 with the drug resistant I223R substitution does contain an opened 150-loop, and consequently the 150-cavity (van der Vries et al., 2012). When scrutinized, in the 09N1-I223R structure, phosphate is also present and a salt bridge is formed between the phosphate ion and the K150 amino group, which maintains the open 150-loop (van der Vries et al., 2012) (Fig. 1C). The structures of 09N1 and its mutant 09N1- I149V fit well with each other with a RMSD of 0.097 Å (Fig. 1D).

**Figure 1:**
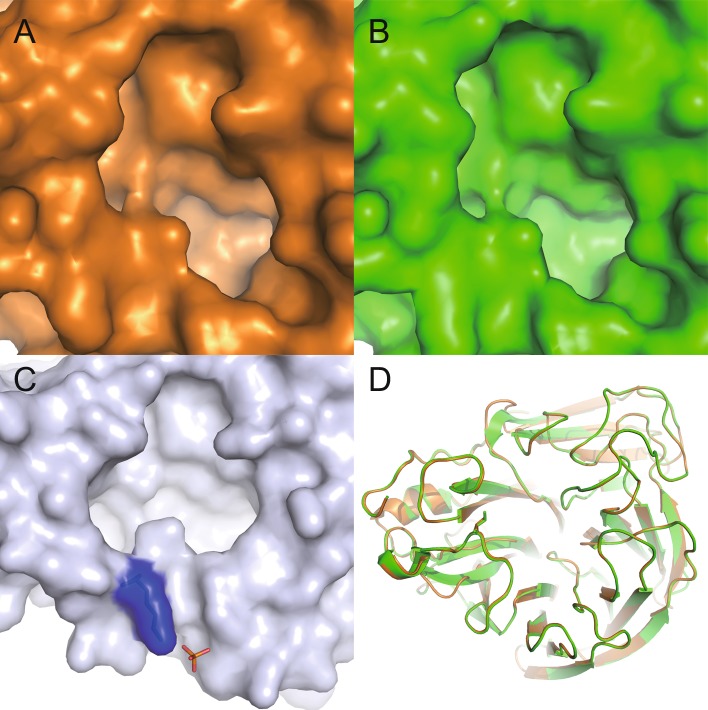
**Comparison of the enzymatic active site of 09N1 and its mutant 09N1-I149V**. (A and B) Surface representation of the active site of 09N1-I149V (orange) and 09N1 (green), respectively; (C) Surface representation of 09N1-I223R active site (white), where a phosphate ion (orange) interacts with K150 (blue) to open up the 150-cavity; (D) Alignment of 09N1 and 09N1-I149V in cartoon representations. 09N1 is in green and 09N1-I149V is in orange; The RMSD between the two structures is only 0.097 Å

Previous molecular dynamics simulations have concluded that the 150-loop of 09N1 should prefer the open conformation and that the I149V mutant should prefer the closed conformation (Amaro et al., 2011). Yet, we can directly observe in high resolution crystal structures that both 09N1 and the I149V mutant prefer a closed 150-loop conformation, relative to other group 1 NAs, specifically N5 and N8 (Figs. 2 and S1).

**Figure 2:**
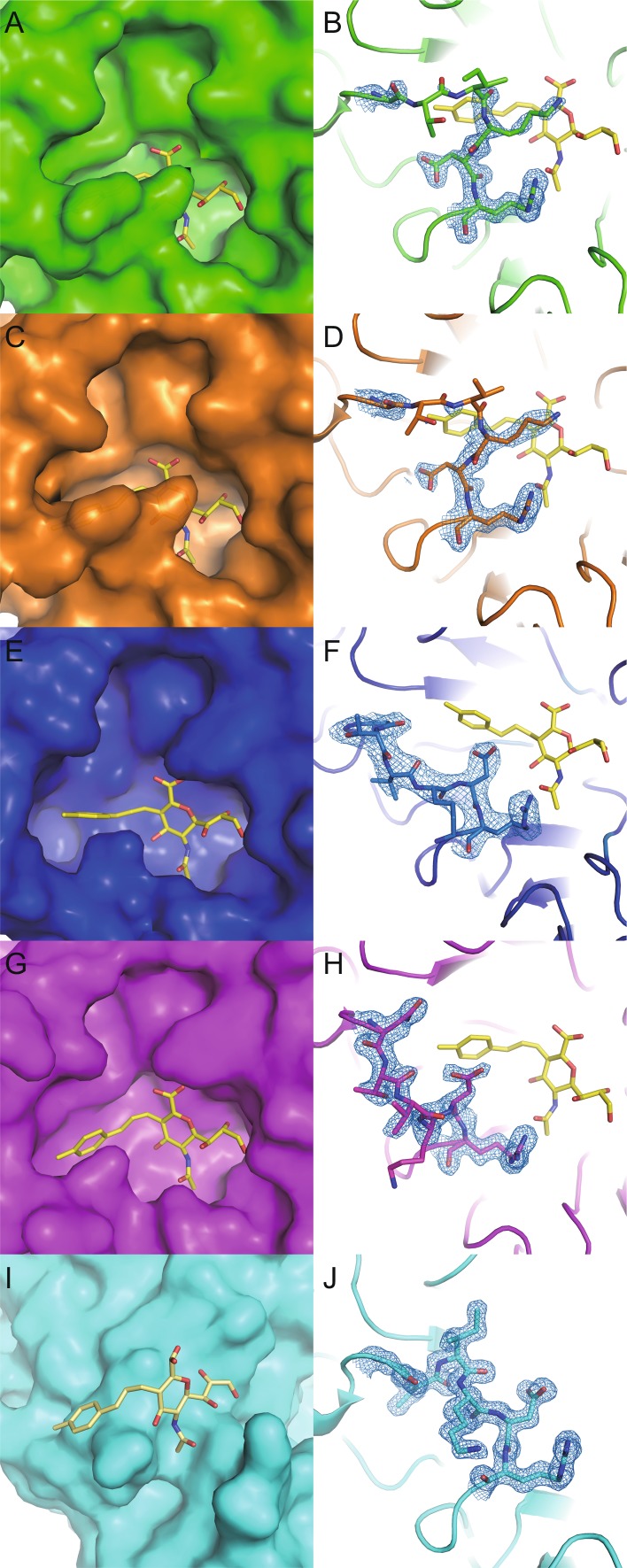
**3-(** ***p*** **-Tolyl)allyl-Neu5Ac2en is a group 1 specific inhibitor**. The left column shows the molecular surface of 09N1 (A, green), 09N1-I149V (C, orange), N8 (E, blue), N5 (G, magenta) and N3 (I, cyan), all in complex with 3-(*p*-tolyl)allyl-Neu5Ac2en, which is shown in stick representation. The right column is the corresponding sigma A weighted electron density map contoured at 1 sigma for the 150-loop in cartoon representation (B, 09N1; D, 09N1-I149V; F, N8; H, N5 and J, N3). For the group 2 NA N3, 3-(*p*-tolyl)allyl-Neu5Ac2en binds to the second sialic acid binding site instead of the active site

The complex structures of 3-(*p*-tolyl)allyl-Neu5Ac2en with 09N1 and 09N1-I149V were solved at 1.7 Å and 2.0 Å, respectively. In both complex structures, the atypical group 1 150-loops are forced half-open into a similar disordered conformation with electron density lacking for residues T148 and I149 (Fig. 2A–D). This explains why the atypical group 1 09N1 can still be inhibited by 150-cavity targeting inhibitors, but with lower efficacy (Rudrawar et al., 2010), as the 150-loop is flexible enough to accommodate the inhibitors.

The complex structure of N5 with 3-(*p*-tolyl)allyl-Neu5Ac2en was solved at 1.8 Å and the complex structure clearly showed the characteristics observed earlier for the N8 complex (Rudrawar et al., 2010) (Fig. 2E–H). Soaking of group 2 NAs, N2 and N3, with 3-(*p*-tolyl)allyl-Neu5Ac2en did not result in typical NA inhibitor complex structures, with the inhibitor bound in the active site. Rather, for N3, 3-(*p*-tolyl)allyl-Neu5Ac2en was bound to the second sialic acid binding site between two adjacent NA molecules (Fig. 2I and 2J).

In our experiments, the inability to obtain complex structures with group 2 N2 or N3 supports the hypothesis that the inhibitor 3-(*p*-tolyl)allyl-Neu5Ac2en exhibits specificity for group 1 NA. This explains further why poorer inhibition of N2 by this group-1-targeted inhibitor was generally observed (Rudrawar et al., 2010). From the aspect of drug-resistance, 3-(*p*-tolyl)allyl-Neu5Ac2en, which is based upon Neu5Ac2en, has an advantage over other group 1 inhibitors which are based upon the oseltamivir structure (Kerry et al., 2013).

To further the biological relevance of our data, we evaluated the inhibition efficacy of 3-(*p*-tolyl)allyl-Neu5Ac2en to both 09 pH1N1 and 09 pH1N1-I149V viruses in cell-based plaque reduction assay. Recombinant viruses were generated by reverse genetics using previously described methods (Wu et al., 2013). The IC_50_ values were determined by using the same titer of 09 pH1N1 and 09 pH1N1-I149V viruses. 4-MUNANA (4-methylumbelliferyl-N-acetylneuraminic acid) was used as a fluorescent NA substrate according to previously reported methods (Wu et al., 2013). As expected, the efficacy of 3-(*p*-tolyl)allyl-Neu5Ac2en for virus is in micro molar range (the corresponding IC_50_ values for 09 pH1N1 and the I149V mutant virus were 35 µmol/L and 31 µmol/L, respectively), which needs to be improved for a clinically-therapeutic inhibitor. Therefore, with this work we provide further evidence of the binding modes that are possible for the novel class of NA inhibitor represented by 3-(p-tolyl)allyl-Neu5Ac2en, which will contribute to further development of this inhibitor class in the future.

In conclusion, our results indicate that 09N1 is truly an atypical group 1 NA and possesses unique 150-loop properties, but the group 1 specific inhibitors still do work against it. Furthermore, the novel complex structures in this study demonstrate the inherent flexibility of the group 1 150-loop, which can adopt many distinct conformations. Therefore it is probable that more structural diversity will be observed in future structural studies. In addition, we also provided the crystal-clear evidence that the novel inhibitor can bind the group 2 member N3 in its second sialic acid binding site. The biological significance of this observation is worthy of further investigation.

## Electronic supplementary material

Supplementary material 1 (PDF 415 kb)
