# Supplementary material for: Atypical group 1 neuraminidase pH1N1-N1 bound to a group 1 inhibitor
Source: Protein Cell. 2015 Sep 3;6(10):771–3. doi: 10.1007/s13238-015-0197-6 (PMC4598326; doi:10.1007/s13238-015-0197-6)
Supplement: Supplementary file 1 — Supplementary material 1 (PDF 415 kb) [file 13238_2015_197_MOESM1_ESM.pdf]

Supplementary Figure 1 Sigma A weighted 2Fo-Fc electron density map for 3-(*p*-tolyl)allyl-Neu5Ac2en bound to different subtype NAs. The following color scheme is used: A, 09N1 (green), B, 09N1-I149V mutant (orange), C, N8 (blue), D, N5 (magenta) and E, N3 (cyan). The maps are contoured at 1.5 sigma,or only 1 sigma for N8 (C) and N3 (E).

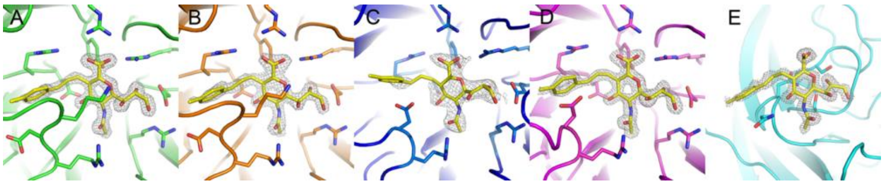

Supplementary Table 1 Data collection and refinement statistics

	09N1-I149V	09N1_IG173	09N1-I149V_IG173	N3_IG173	N5_IG173
**Datacollection**					
Spacegroup	$C222_1$	$C222_1$	$C222_1$	I4	P4
Celldimensions					
a,*b*,*c*(Å)	118.17,136.54,117.89	118.93,138.39,118.88	117.59,136.29,117.65	106.05,106-05,66.41	112.66,112.66,
α, β, γ (°)	90,90,90	90,90,90	90,90,90	90,90,90	90,90,90
Resolution(Å)	50-1.60(1.66-1.60)	50-1.70(1.76-1.70)	50-2.00(2.07-2.00)	50-1.60(1.66-1.60)	50-1.80(1.86-1
R_{merge}	0.086(0.395)	0.162(0.467)	0.12.7(0.552)	0.082(0.135)	0.113(0.461)
$I/\sigma I$	25.2(7.8)	11.7(2.1)	15.3(3.9)	24.7(11.4)	18.7(5.0)
Completeness (%)	97.9(96.4)	97.5(86.4)	99.9(99.9)	100.0(99.7)	100.0(100.0)
Redundancy	6.6(6.5)	6.8(4.6)	6.3(6.1)	7.2(4.7)	6.9(7.1)
**Refinement**					
Resolution(Å)	32.79-1.60	36.29-1.70	49.08-2.00	33.54-1.60	43.03-1.80
No.reflections	121574	104138	60452	48212	75083
R_{work}/R_{free}	0.1429/0.1612	0.1560/0.1817	0.1556/0.1875	0.1313/0.1529	0.1492/0.1795
No.atoms					
Protein	6090	6046	6044	3101	6158
Ligand/ion	5	65	65	32	64
Water	918	943	556	538	845
B-factors					
Protein	11.3	10.0	20.3	7.9	14.3
Ligand/ion	10.1	13.9	23.5	10.9	12.7
Water	30.3	26.5	30.5	23.8	28.9
R.m.s.deviations					
Bondlengths(Å)	0.007	0.009	0.009	0.008	0.006
Bondangles(°)	1.176	1.243	1.230	1.244	1.062

*Values in parentheses are for highest-resolution shell. Here 3-(p-tolyl)allyl-Neu5Ac2en is referred to as IG173.
